# Electrocardiographic Markers of Atrial Cardiomyopathy: Strengths and Limits of P‐Wave–Based Assessment

**DOI:** 10.1111/anec.70152

**Published:** 2026-01-09

**Authors:** Mehmet Mustafa Yılmaz, Mücahit Aker, Macit Kalçık

**Affiliations:** ^1^ Department of Cardiology Hitit University Erol Olçok Education and Research Hospital Corum Turkey; ^2^ Department of Cardiology, Faculty of Medicine Hitit University Corum Turkey

To the Editor,

We read with interest the article by Kazantzi et al. examining the association between established P‐wave parameters and left atrial hemodynamics in the context of atrial cardiomyopathy (ACM) (Kazantzi et al. 2026). The authors should be acknowledged for addressing a clinically relevant question using a prospective design and comprehensive echocardiographic assessment. Their conclusion that advanced interatrial block (IAB) is the most reliable electrocardiographic marker of impaired left atrial function is clearly presented and supported by internal consistency within the dataset.

Nevertheless, several methodological aspects warrant closer scrutiny. The study population is heterogeneous, comprising patients with prior atrial fibrillation, embolic stroke of undetermined source, and individuals without manifest cardiovascular disease. While this broad inclusion enhances external validity, it may dilute pathophysiologically specific associations between P‐wave indices and atrial remodeling. Current consensus documents emphasize that ACM represents a spectrum with variable electrical, structural, and mechanical manifestations, and pooling such diverse phenotypes may obscure parameter‐specific diagnostic performance (Goette et al. 2024).

A further concern relates to the operational definition of pathological P‐wave parameters. Thresholds derived from consensus recommendations are largely based on epidemiological associations rather than mechanistic validation against atrial tissue pathology or gold‐standard imaging. In particular, the dismissal of P‐wave terminal force in lead V1 as a hemodynamic marker may reflect cohort characteristics rather than a true lack of biological relevance, given its previously demonstrated association with atrial fibrosis and stroke risk in population‐based studies (Kamel et al. 2014).

Additionally, the reliance on cross‐sectional echocardiographic markers limits causal inference. Left atrial strain and PA‐TDI are sensitive indicators of atrial function, yet they remain load‐dependent and subject to inter‐vendor variability. Longitudinal assessment or correlation with advanced imaging modalities such as late gadolinium‐enhanced cardiac magnetic resonance imaging could have strengthened the argument that advanced IAB truly captures the substrate of atrial cardiomyopathy rather than representing an epiphenomenon of aging and comorbidity burden (Bisbal et al. 2020).

Finally, the clinical implications of prioritizing advanced IAB over other P‐wave parameters deserve cautious interpretation. While advanced IAB appears strongly associated with impaired atrial hemodynamics, its relatively low prevalence may limit utility as a screening tool. A multiparametric approach integrating electrocardiographic, echocardiographic, and biomarker data may better reflect the complex biology of ACM and align with contemporary views of atrial disease as a continuum rather than a binary entity (Kreimer and Gotzmann 2022).

## Author Contributions

All of the authors contributed to planning, writing, and revision.

## Funding

The authors have nothing to report.

## Conflicts of Interest

The authors declare no conflicts of interest.

## Data Availability

Data sharing not applicable to this article as no datasets were generated or analyzed during the current study.
